# Beyond the Barriers of Ex Vivo Lung Perfusion Through an Emblematic Case: A New Way Forward to Expand the Donor Pool

**DOI:** 10.3390/jcm13237412

**Published:** 2024-12-05

**Authors:** Eleonora Faccioli, Vincenzo Verzeletti, Marco Mammana, Andrea Dell’Amore, Luca Melan, Fares Shamshoum, Edoardo Rosellini, Annalisa Boscolo, Federica Pezzuto, Paolo Navalesi, Fiorella Calabrese, Federico Rea, Marco Schiavon

**Affiliations:** 1 Thoracic Surgery Unit, Department of Cardiac, Thoracic, Vascular Sciences and Public Health, University of Padua, 35128 Padua, Italy; vincenzo.verzeletti@aopd.veneto.it (V.V.); marco.mammana@aopd.veneto.it (M.M.); andrea.dellamore@unipd.it (A.D.); luca.melano@aopd.veneto.it (L.M.); fares.shamshoum@aopd.veneto.it (F.S.); federico.rea@unipd.it (F.R.); marco.schiavon@unipd.it (M.S.); 2 Anesthesia and Intensive Care Unit, University Hospital of Padua, 35128 Padua, Italy; edoardo.rosellini@aopd.veneto.it (E.R.); annalisa.boscolobozza@unipd.it (A.B.); paolo.navalesi@unipd.it (P.N.); 3 Department of Medicine, University of Padua, 35122 Padua, Italy; 4 Department of Cardiac, Thoracic, Vascular Sciences and Public Health, University of Padua, 35122 Padua, Italy; 5 Pathology Unit, Department of Cardiac, Thoracic, Vascular Sciences and Public Health, University of Padua, 35122 Padua, Italy; federica.pezzuto@unipd.it (F.P.); fiorella.calabrese@unipd.it (F.C.)

**Keywords:** EVLP, lung transplant, PGD, machine perfusion, marginal donor

## Abstract

**Background:** Lung transplantation is the most effective treatment for end-stage respiratory diseases, but its application is limited by the shortage of organs. Ex vivo lung perfusion (EVLP) has emerged as a promising technique to evaluate and recondition donor lungs previously deemed unsuitable for transplantation. However, limitations such as lung contusions, air leaks, and perfusate extravasation, especially in portable EVLP systems, hinder the procedure. Despite prolonged perfusions that can result in blood pooling at the lung bases due to fixed lung positioning and diminished oncotic pressure, in some cases, extending perfusion time beyond the typical 5–6 h could benefit extended-criteria lungs, addressing factors such as edema or logistical complications. **Methods:** We present an innovative protocol involving prolonged EVLP, pronation of the graft, and the addition of anti-edematous drugs to the perfusate. **Results:** This novel approach, previously tested in animal models, enhances lung reconditioning and expands the donor pool. **Conclusions:** Our findings suggest that this strategy overcomes key limitations of standard EVLP, offering a valuable solution for improving the availability of transplantable lungs.

## 1. Introduction

Lung transplantation remains the most effective treatment for patients with end-stage respiratory disease. However, its widespread application is hindered by a significant shortage of available organs [1].

In this challenging context, ex vivo lung perfusion (EVLP) has emerged as a promising technique for evaluating and reconditioning donor lungs that might have previously been deemed unsuitable for lung transplantation [1,2]. This technique has shown encouraging results both with static and portable platforms [3,4] and in the setting of extended-criteria donors [5].

Despite its potential, the initiation of an ex vivo procedure is limited by the presence of lung contusions, which can lead to air leaks and perfusate extravasation, thereby restricting the effectiveness of the perfusion technique. Additionally, in the portable EVLP systems that utilize a cellular perfusate, prolonged perfusions often result in blood pooling at lung bases. This may be due to the fixed positioning of the lungs during the procedure and the diminishing oncotic power of the dextrane-based perfusate over time.

Nevertheless, extending the usual perfusion time of 5–6 h could be crucial in several cases involving extended-criteria lungs for various reasons. Firstly, several organ alterations, such as edema, infections, or suspicion of aspiration, may require longer reconditioning and monitoring time. Secondly, logistic issues such as procurement complications and long distances between donor and recipient hospitals may benefit from extended preservation [6]. In addition to prolonged perfusion, we have observed that extended-criteria lungs can also benefit from invasive maneuvers during EVLP, such as pronation. This technique can help better perfuse and ventilate the most declivous areas of the grafts. Furthermore, the addition of specific drugs to the perfusate can improve organ conditions, making them suitable for transplantation.

Based on these considerations, we would like to share insights about an “unusual” prolonged use of EVLP, combined with a new protocol, previously tested only on animal models, that includes graft pronation and the addition of anti-edematous drugs to the perfusate. This strategy effectively addresses the previously reported limitations of the procedure in order to expand the donor pool.

## 2. Case Presentation

We present a recent experience in which organs not deemed suitable for transplant by several other centers were successfully transplanted after prolonged normothermic perfusion, a cycle of pronation, and the correction of graft edema by adding high doses of albumin.

The donor was a 34-year-old brain-dead female who suffered a subarachnoid hemorrhage. She had a mild smoking history (5 packs/year) and was located in a hospital on an Italian island more than 1300 km away from our center, with an estimated transport time of more than 6 h. The donor had been hospitalized in the ICU for 2 days prior to procurement. The organs were initially judged to be low-quality due to the presence of abundant fluid secretions at the bronchoscopy upon arrival at the hospital, a low PO_2_/FiO_2_ (P/F) ratio of 290 mmHg at the moment of the organ offer, and the presence of edema and contusions from cardiopulmonary resuscitation maneuvers revealed at the retrieval, resulting in an OTO score of 9 [7]. The recipient was a 48-year-old man suffering from chronic respiratory failure related to hypersensitivity pneumonitis.

Considering the donor’s young age, the improvement in bronchoscopy findings during ICU hospitalization and the possibility for lung reconditioning with EVLP, we decided to retrieve the lungs and transport them to our center with the OCS Lung™. To mitigate pulmonary edema, we decided to add a 250 mL 5% albumin solution from the beginning alongside the standard drugs. To avoid excessively increasing the perfusate volume with the addition, we primed the machine with 1.5 L of OCS solution (generally, a volume between 1.5 L and 2 L is allowed).

At the first functional assessment, the P/F ratio was 366 mmHg, vascular resistances were 1024 dyn.s/cm^5^, and the mean pulmonary arterial pressure was 5 mmHg. Upon visual inspection, the lungs were still mildly edematous with heavy bases and moderate foamy secretions detected at the first bronchoscopy check.

**Figure 1:**
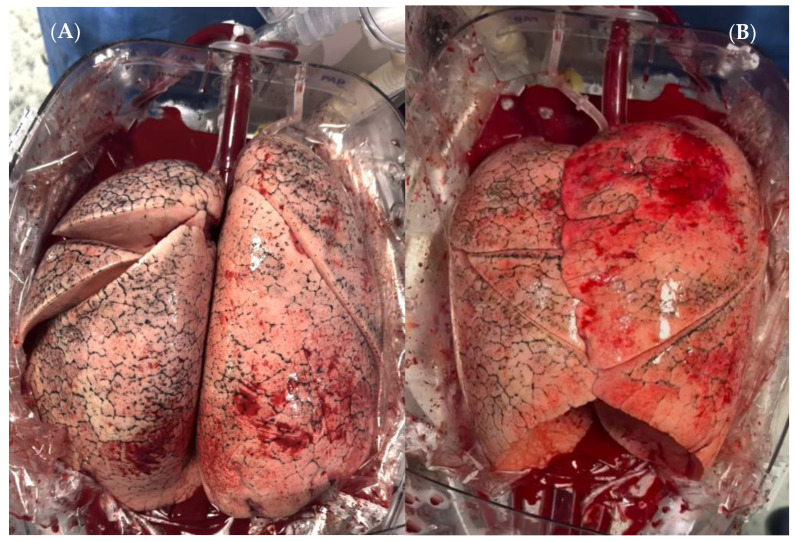
(**A**) pronated lungs; (**B**) supinated lungs during EVLP.

The perfusion lasted 9 h and 6 min and was conducted with low pulmonary artery flow (up to 1.5 L/min) and under protective ventilation (PEEP 5 cm H_2_O, tidal volume of 5 mL/Kg). After 6 h of perfusion, once the lungs arrived at the recipient hospital, the organs were pronated for 60 min (Figure 1A). To perform this, we first clamped the tracheal cannula, disconnecting it from the ventilation. Then, we gently rotated the arterial line, trying to avoid kinking in the pressure line, and simultaneously rotated the lungs, reconnecting them to the ventilator. This method allowed us to avoid issues related to the open outflow system, as the blood smoothly flowed to the reservoir without compromising continuous organ evaluation.

All these measures resulted in a progressive lightening of the lung bases, reduction in lung edema, and improvement in gas exchanges (last P/F ratio was 493 mmHg). During the reconditioning, we performed a total of three bronchoscopies, which also showed a progressive marked reduction in the quantity of the foamy secretions, which were not detectable by the last check. We also performed an X-ray of the grafts during the EVLP, which did not show direct signs of lung edema. The trend of all cardiovascular and respiratory parameters available during the OCS run is reported in Figure 1B. The P/F ratio showed substantial improvement (from 290 mmHg to 493 mmHg); the mean pulmonary artery pressure and the vascular resistance remained stable (between 4 and 6 mmHg and 200 and 600 dyn·s/cm^5^·m^2^, respectively). The respective peaks at six hours of perfusion could be attributed to the pronation, which may cause a kinking in the arterial line. Additionally, the peak airway pressure trend improved during the ex vivo perfusion (from 16 cm H_2_O at the beginning to 6 cm H_2_O at the end). For all these reasons, we considered the lungs suitable for transplantation.

**Figure 2:**
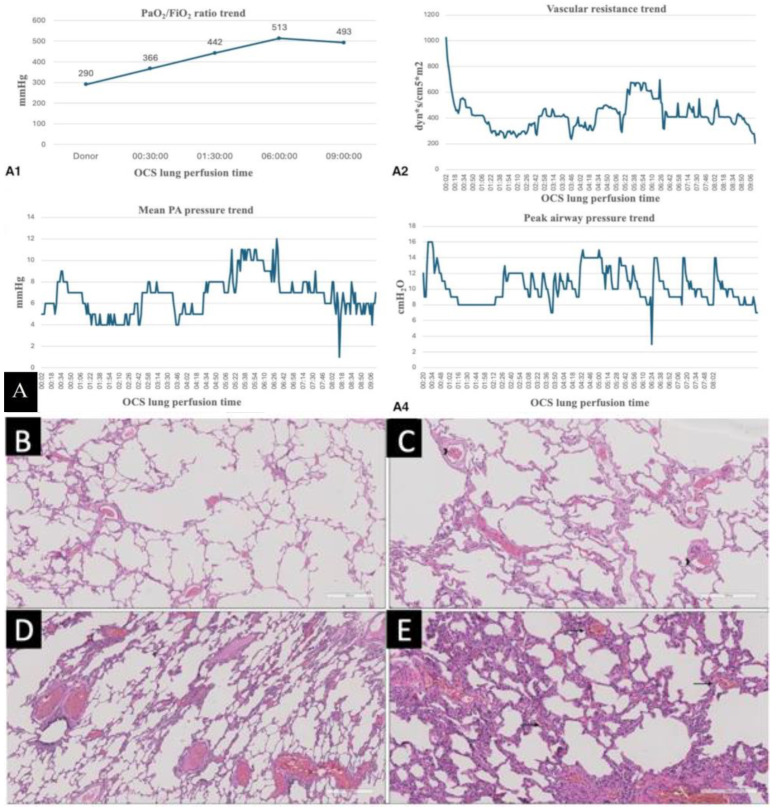
(**A**) OCS lung parameters during normothermic perfusion showing an increase in the PaO_2_/FiO_2_ ratio, a reduction in vascular resistance trend, and stability of peak airway pressure and pulmonary pressure; (B) histological examination of the pre-reperfusion biopsy (hematoxylin and eosin, scale bar: 600 µm); (**C**) higher magnification of the histological examination of the pre-reperfusion biopsy (hematoxylin and eosin, scale bar: 300 μm. Arrowhead on lymphocytes); (**D**) histological examination of the post-reperfusion biopsy (hematoxylin and eosin, scale bar: 500 μm); (**E**) higher magnification of the histological examination of the post-reperfusion biopsy (hematoxylin and eosin, scale bar: 300 µm. Arrows on neutrophilic margination).

The bilateral lung transplant was conducted with central veno-arterial extracorporeal membrane oxygenation (ECMO) through a clamshell approach, according to our standard protocol, without complications. The assistance was removed about 30 min after reperfusion of the second lung, given the excellent gas exchanges and satisfactory hemodynamics. The surgery lasted 5 h and 5 min without any intraoperative complications. No volumetric graft reductions were performed, given the correct size matching between donor and recipient. To evaluate the possible ischemia–reperfusion damage induced by the prolonged reconditioning during transplantation, we also collected pre- and post-reperfusion biopsies (Figure 2B–E).

In the pre-reperfusion biopsy, histological examination revealed mild congestion (score 1) without accompanying edema (score 0). Notably, a focal alveolar septa rupture was observed. At higher magnification, scattered lymphocytes within small vessels were noted, while neutrophil margination was absent (score 0).

The post-reperfusion biopsy exhibited moderate congestion (score 2), concomitant with emphysematous changes. An increase in neutrophilic margination, graded as moderate (score 2), was accompanied by alveolar septal thickening due to vascular dilatation and inflammation.

No primary graft dysfunction (PGD) was detected at 0, 24, 48, and 72 h, and the patient was extubated on post-operative day (POD) 1. The ICU stay lasted 4 days. The patient experienced one post-operative complication (the onset of atrial fibrillation on POD 10), which was treated with pharmacological cardioversion.

Transbronchial biopsies performed on POD 25 showed no signs of rejection, and the patient was discharged on POD 31.

## 3. Discussion

In recent years, several innovative strategies have been developed to expand the donor lung pool, including advancements in donor management, the utilization of lungs from donors after cardiac death (DCD), the use of lobar lung transplants from living donors (LLLD), and the implementation of EVLP for assessing and reconditioning extended-criteria donors.

The potentiality of portable and static EVLP platforms in expanding the donor pool has already been well described in the current literature [3] and in cases where prolonged perfusions are necessary [5].

At our center, we have progressively gained experience with the portable machine perfusion system (OCS Lung). Since our first case performed in 2012, we have successfully perfused and implanted over 50 grafts. This device is currently the only one approved by the Food and Drug Administration for portable normothermic EVLP, and it relies on normothermic, blood-based perfusion, ventilation, monitoring, and recruitment [3,8]. Basically, it overcomes the issue of cold ischemic time, maintaining the organ in a physiologic state during transport. To provide a basic explanation of the device’s mechanism, it starts with a pulmonary artery cannula connected to the pulmonary artery. Additives such as antibiotics, steroids, multivitamins, anti-fungal drugs, and insulin are required to be added to the perfusate. Blood is circulated through the lungs and then passively drains into the left atrium. It then flows into a reservoir, where a pulsatile pump propels it through an oxygenator maintained at a specific temperature. Finally, the blood is returned to the pulmonary artery, repeating the cycle. The trachea is connected to a miniaturized ventilator, which provides ventilation to the lungs.

We believe that the reported case is innovative for the following reasons: (1) the decision to perform prolonged perfusion despite the presence of contusions, which are a relative contraindication for the EVLP utilization; (2) the addition of albumin to the perfusate, despite the usual priming solution containing dextrane, to maximize the anti-edematous effect of the perfusate; (3) the pronation of the grafts in the OCS Lung™.

The optimal composition of the perfusate is a critical area of research in ex vivo lung perfusion, particularly for optimizing lung quality during prolonged procedures. Animal studies have explored various perfusates (cellulate vs. acellulate) during 24 h OCS perfusion to identify which yields the best outcomes [9]. Among these, cellular STEEN solution, supplemented with erythrocytes, demonstrated superior post-transplant survival and oxygenation.

Regarding pronation, one of the main concerns with machine perfusion devices is the supine positioning of the lungs during perfusion, which may lead to a mismatch in ventilation-perfusion distribution; this can increase vascular permeability and contribute to pulmonary edema. Based on this concern, several authors have demonstrated the benefits of prone positioning in porcine lungs with static EVLP perfusion. Nikawa et al. [10] showed that the pronation group exhibited less ischemia–reperfusion injury and better lung function. The same authors subsequently reported successful lung transplantation following the pronation of human lungs during static EVLP perfusion [11].

The pronation of human lungs on the portable platform has not previously been described. We believe that this procedure should be performed by trained surgeons, as it carries potential risks: if pronation is not performed accurately, there is a risk of arterial line or tracheal disconnection, which could result in perfusate loss or lung deflation, respectively. Additionally, improper pronation may lead to kinking of the pulmonary artery, potentially increasing mean pulmonary artery pressure and vascular resistance, potentially worsening the organ’s quality during ex vivo procedures.

In these emblematic cases, the adopted innovations, combined with a slow and controlled recruitment of the lungs, contributed to making the transplantation possible by utilizing these lungs, which would have been missed without the reconditioning and extensive evaluation with machine perfusion. In the absence of previous experience with this protocol, the dose of the albumin added to the perfusate and the duration of the pronation were established in accordance with the manufacturing scientists and engineers, following an animal experimental model using this protocol. Naturally, it should be specified that deviations from the standard procedure, such as those described above, should only be performed by transplant teams with extensive expertise in organ perfusion techniques.

## 4. Conclusions

In conclusion, we believe that this deviation from the standard protocol should become routine practice when reconditioning lungs in centers with extensive expertise in portable EVLP device utilization, particularly for improving lung quality during prolonged perfusions. Investing resources and knowledge in this field presents a promising strategy to address the ongoing issue of organ shortages.

## Data Availability

The raw data supporting the conclusions of this article will be made available by the authors upon request.
